# Pathogenesis of Renal Failure in Multiple Myeloma: Any Role of Contrast Media?

**DOI:** 10.1155/2014/167125

**Published:** 2014-04-30

**Authors:** Michele Mussap, Giampaolo Merlini

**Affiliations:** ^1^Laboratory Medicine Service, IRCCS AOU San Martino-IST, National Institute for Cancer Research, University-Hospital San Martino, Largo Rosanna Benzi 10, 16132 Genova, Italy; ^2^Amyloidosis Research and Treatment Center, Clinical Chemistry Laboratories, Fondazione Istituto di Ricovero e Cura a Carattere Scientifico (IRCCS) Policlinico San Matteo, Viale Golgi 19, 27100 Pavia, Italy; ^3^Department of Molecular Medicine, University of Pavia, Italy

## Abstract

The spectrum of kidney disease-associated monoclonal immunoglobulin and plasma cell malignancies is remarkably broad and encompasses nearly all nephropathologic entities. Multiple myeloma with kidney impairment at presentation is a medical emergency since the recovery of kidney function is associated with survival benefits. In most cases, kidney impairment may be the first clinical manifestation of malignant plasma cell dyscrasias like multiple myeloma and light chain amyloidosis. Multiple myeloma per se cannot be considered a main risk factor for developing acute kidney injury following intravascular administration of iodinated contrast media. The risk is increased by comorbidities such as chronic kidney disease, diabetes, hypercalcemia, dehydration, and use of nephrotoxic drugs. Before the administration of contrast media, the current recommended laboratory tests for assessing kidney function are serum creatinine measurement and the estimation of glomerular filtration rate by using the CKD-EPI equation. The assessment of Bence Jones proteinuria is unnecessary for evaluating the risk of kidney failure in patients with multiple myeloma, since this test cannot be considered a surrogate biomarker of kidney function.

## 1. Introduction

The kidney is susceptible to insult from a variety of exogenous (e.g., drugs, organic compounds) and endogenous (toxins, catabolites, etc.) substances because of its complex anatomical, physiological, and biochemical features. Factors contributing to the sensitivity of the kidney include its large blood flow (20–25% of resting cardiac output corresponding to approximately 1.2-1.3 L/min), concentration of filtered solutes during urine production (kidneys reabsorb more than 99% of the glomerular filtrate), and the presence of a variety of xenobiotic transporters and metabolizing enzymes. The list of the potential nephrotoxic substances includes drugs, environmental agents, heavy metals, radiocontrast agents, and immunoglobulin free light chains (FLCs). Each of them may adversely affect one or more sites along the nephron, leading to a progressive organ damage and impairment named toxic nephropathy [1]. Toxic nephropathy commonly occurs either as a result of decreased renal perfusion or as direct toxic effects on the proximal tubule; both mechanisms originate specific clinical syndromes (e.g., proximal tubule necrosis, hyperkalemia, medullary thick ascending limb injury). Toxic nephropathy originating from renal or systemic diseases (e.g., nephrotic syndrome, multiple myeloma) can be drastically worsened by such additional conditions (e.g., dehydration, hypoxia), as well as by the effects of potentially nephrotoxic substances (e.g., drugs, contrast media) administered in the course of the disease. The latter condition calls for a careful monitoring of patients in order to avoid the risk of a subsequent nephrotoxic insult leading to severe kidney diseases, like acute tubular necrosis (ATN) and, more extensively, acute kidney injury (AKI).

## 2. Kidney Diseases Associated with Plasma Cells Dyscrasias

Monoclonal gammopathies refer to a spectrum of disorders characterized by the monoclonal proliferation of lymphoplasmacytic cells in the bone marrow and, sometimes, tissue deposition of monoclonal immunoglobulins (Igs) or their components [2]. The most frequent diseases include monoclonal gammopathy of undetermined significance (MGUS), both asymptomatic (smoldering) and symptomatic multiple myeloma (MM), solitary plasmacytoma, and immunoglobulin light chain amyloidosis (AL amyloidosis) [3]. In patients with MM, the incidence of kidney disease throughout the course of the disease ranges between 15 and 40%, depending on the definition of kidney injury and failure [4, 5]. Kidney impairment may be the first clinical manifestation of MM and related conditions, such as light chain amyloidosis [6]. Despite a high rate of partial and complete hematological response of MM to individually tailored therapies in the novel agent era [7], renal recovery is still poor [8]. MM with renal impairment at presentation should be considered a medical emergency since the recovery of renal function is associated with survival benefit [9, 10]. The spectrum of kidney disease associated with monoclonal gammopathies is remarkably broad and encompasses nearly all nephropathologic entities (Table 1). The three most common forms of monoclonal immunoglobulin-mediated kidney disease are cast nephropathy, monoclonal immunoglobulin deposition disease (MIDD), and AL amyloidosis. A growing number of pathologic renal conditions are being attributed to a clonal plasma cell disorder that is less “myeloma-like” and more “MGUS-like” in terms of its bulk and proliferative rate [11–13]. Uncommon conditions reported in Table 1 have been recently grouped in the new entity of monoclonal gammopathy of renal significance (MGRS) with the intent to discriminate the pathologic nature of these diseases from the truly benign MGUS [14]. The tubulointerstitial injury, cast nephropathy, is the most common cause of severe AKI in patients with MM (≅90% of cases). Data both from the US Renal Data System and from the European Renal Association-European Dialysis and Transplant Association Registry reveal that 1.5% of patients placed on renal replacement therapy have MM; in patients with end stage renal disease (ESRD) due to MM, the mortality rate is 58% compared with 31% in all other ESRD patients [15–17]. Despite the high heterogeneity of kidney disease in plasma cell dyscrasias, the risk of progression to severe organ impairment leading to renal replacement therapy is considerable; thus, it is mandatory to identify patients at risk for kidney damage at a very early stage and to institute treatment promptly. In addition, there is a need for accurate monitoring of these patients to avoid or at least to delay the development of ESRD and dialysis.

## 3. Pathogenesis of Kidney Failure in Malignant Plasma Cells Dyscrasias

In MM, the high monoclonal serum FLCs concentration results in a burden of FLCs on the proximal tubule that overwhelms the capacity of both the megalin and cubilin receptors to reabsorb the FLCs. As a consequence, large amounts of FLCs reach the distal tubule lumen where they interact specifically with Tamm-Horsfall proteins (THPs; also known as uromodulin), generating myeloma casts. Cast formation in the distal tubule can block glomerular flow and cause proximal tubular atrophy [19], also contributing to interstitial fibrosis [20]. Simultaneously, the massive reabsorption of monoclonal FLCs within the proximal tubules induces proximal tubule cells apoptosis and DNA degradation, resulting in critical morphologic changes, such as epithelial-to-mesenchymal transition (EMT) or necrosis [21]. In addition, FLCs activate a sequence of inflammatory cascade through nuclear transcription factors, nuclear factor kappa B (NF*κ*B), and AP-1 (c-fos and c-jun) complexes. These transcription factors induce in turn the synthesis of the proinflammatory cytokines interleukin-6 (IL-6), macrophage chemoattractant protein-1 (MCP-1), and tumor necrosis factor *α* (TNF*α*) and activate signaling pathways, such as mitogen-activated protein kinases (MAPK), extracellular signal regulated kinase (ERK1/2), Jun kinase (JNK), and p38 MAPK [22]. As a result, the peculiar histologic lesion of the myeloma cast nephropathy consists of a chronic tubulointerstitial nephropathy with marked tubular atrophy, laminated intratubular casts, and extensive interstitial fibrosis. Ultimately, renal fibrogenesis is a major mechanism of kidney impairment in multiple myeloma [23].

## 4. Mechanisms of Free Light Chains Nephrotoxicity

The risk of AKI in patients with MM increases with the concentration of FLCs in urine; however, not all monoclonal FLCs are nephrotoxic. Nephrotoxicity appears to be an intrinsic property of some FLCs, as indicated by the recurrence of similar renal lesions after kidney transplantation [24, 25]. From a clinical point of view, this is confirmed by the fact that a number of patients with considerable FLCs proteinuria never develop kidney disease [26]. As FLCs are composed of different amino acids, they have different isoelectric points (pIs) in solution due to the differences in electrical charge of individual amino acids. As the pH of the solution reaches that of the protein pI, the net protein electrical charge approaches zero and as such protein precipitation becomes more likely. In kidney disease induced by monoclonal FLCs, the pattern of kidney injury depends on both structural peculiarities of monoclonal FLCs, particularly of the variable (V) domain, and environmental factors, such as pH, urea concentration, or local tissue proteolysis. Moreover, intrinsic host factors play a pivotal role in determining both the type and severity of any renal response to a given FLC. The variable (V) region of the immunoglobulin light chains comprises four key sections that come together to form a hydrophobic core and to allow for the variety of antigen binding, containing three highly variable segments, termed complementary determining regions (CDR), which are attached to joining sections (J). As such *κ* light chains comprise 40 V*κ* and 5 J*κ* sections, whereas *λ* light chains have 30 V*λ* and 8 J*λ*. Although designed to bind antigens, the presence of tyrosine and tryptophan allows additional binding possibilities to lipid-rich cell membranes by binding to sphingomyelin [27, 28]. The CDR3 domain in the V region of both *κ* and *λ* FLCs interacts with THP; the binding affinities of FLCs for THP are related to the amino acid composition of the CDR3 domain [29]. On the other hand, THP has a binding domain for FLCs consisting of nine amino acids [30]. In Fanconi syndrome, monoclonal FLCs are nearly always members of the V*κ*1 subfamily and are derived from only two germ line genes,* IGKV1*-*39 *and* IGKV1*-*33* [31]. The replacement of polar residues by nonpolar or hydrophobic residues in the complementarity determining regions of these monoclonal FLCs can induce resistance of the V*κ* domain to proteolysis, resulting in light chain crystallization [32]. In AL amyloidosis and LCDD, the pathogenic role of V regions is suggested by overrepresentation of the V*λ*6 and V*κ*4 subgroups, respectively, N-glycosylation of the V region, and substitutions of key amino acids induced by somatic mutations that might account for the propensity of certain FLCs to aggregate and influence tropism of deposition [33]. In AL amyloidosis, a role for V sequences is also suggested by the high potential of V domain dimerization* in vitro* and* in vivo* [34]. Unfortunately, there are currently no clinically relevant tools for identifying the potential nephrotoxicity of a specific monoclonal FLC.

## 5. Risk Factors and Correlated Mechanisms Precipitating Kidney Failure

Patients with MM are at an increased risk of kidney disease not only from the kidney injuries and damages due to the primary disease, but also from a number of additional concomitant factors that can substantially contribute to worsen kidney function, leading to AKI, ESRD, and ultimately to renal replacement therapy. A list of these risk factors is reported in Table 2. For example, ATN can be precipitated by dehydration in the presence of FLCs *κ* or *λ* that may deposit in the kidney, as described above. The use of loop diuretics may also contribute to cast formation, leading in turn to serum creatinine levels increase. Loop diuretics are an established risk factor for myeloma kidney. In 1990, it was demonstrated that increasing the concentration of sodium chloride facilitated coprecipitation of Bence Jones proteins with human Tamm-Horsfall glycoprotein* in vitro* [35]. Furosemide promotes intranephronal obstruction by increasing sodium chloride concentration in the distal nephron, the site of cast formation. In a study published in 1992 it was demonstrated that furosemide accelerated in a concentration-dependent manner cast formation and subsequent obstruction of nephrons perfused* in vivo* with cast-forming Bence Jones protein [36]. Vasoconstriction, as a result of hypercalcemia, and decreased blood flow from the kidneys, as a result of the use of nonsteroidal anti-inflammatory drugs (NSAIDs) or aminoglycosides, may also damage the kidneys [37]. Hypercalcemia is the second most common cause of kidney failure in MM [38]. Elevated calcium concentration in renal tubules causes intratubular calcium deposition and vasoconstriction in renal vasculature. The decrease in GFR induces cast precipitation. Hypercalcemia may also lead to nephrogenic diabetes insipidus, which is characterized by antidiuretic hormone (ADH) resistance. With the impairment of renal concentrating ability, polyuria and polydipsia may also develop. Unfortunately, increased diuresis results in hypovolemia and aggravates the prerenal component of renal failure. Hypovolemia induces cast precipitation by increasing FLC concentration in tubule lumen and lower urine flow contributes to intratubular obstruction. NSAIDs are used extensively in patients with multiple myeloma to reduce bone pain. They basically block the production of prostaglandins (PGs), which act as vasodilator hormone, via inhibition of cyclooxygenase enzyme activities (COX-1 and COX-2), leading to renal vasoconstriction and to a reduction in the renal blood flow and GFR. PGs blockage also leads to salt and water retention by the inhibition of chloride reabsorption and ADH. Medullary hypoxia is a major consequence of NSAIDs use, as PGE2 is a major factor in matching medullary oxygen supply and demand. In that respect, papillary necrosis is considered to be caused by critical medullary hypoxia determined by NSAIDs.

## 6. Contrast Media-Induced Nephropathy and Plasma Cell Dyscrasias

In most cases, the onset of contrast induced-acute kidney injury (CI-AKI) consists of a transient nonoliguric form of AKI with serum creatinine levels peaking at 48–72 h after administration of the medium and returning to baseline values within the subsequent ten days [39]. While the threshold level of kidney injury used to define CI-AKI varies across studies, the most commonly employed definition is an increase in the serum creatinine concentration of at least 0.5 mg/dL and/or 25% within 3-4 days of contrast exposure [40]. Appreciable nephropathy is unlikely to develop if the serum creatinine level does not increase by more than 0.5 mg/dL within 24 hours [41]. Some patients develop a chronic reduction in kidney function or a permanent need for renal replacement therapy. A prospective study showed that the percentage change of serum creatinine 12 h after contrast versus the basal value was the best predictor of CI-AKI (*P* < 0.001) [42]. A 5% increase of serum creatinine level yielded 75% sensitivity and 72% specificity, with an area under the curve (AUC) of 0.80 and an odd ratio (OR) of 7.37 for early detection. Furthermore, this 12-hour basal value strongly correlated with the development of renal impairment at 30 days (*P* = 0.002; sensitivity 87%, specificity 70%; AUC 0.85; OR 13.29). In a study using cystatin C as an early marker for AKI, a cutoff cystatin C increase concentration of ≥10% at 24 hours after contrast media exposure was detected in 21.2% of patients and was the best cutoff value for the early identification of patients at risk for CI-AKI with a negative predictive value of 100% and a positive predictive value of 39% [43]. As in other cases of AKI, it appears that, in patients with CKD, cystatin C may be a useful marker for the early diagnosis of CI-AKI. Kidney injury resulting from iodinated contrast is potentially preventable [44]. Past efforts to find effective preventive strategies for CI-AKI have focused on four principal approaches: (a) use of less nephrotoxic contrast agents; (b) provision of preemptive renal replacement therapy to remove contrast from the circulation prior to its filtration at the glomerulus; (c) expansion of the intravascular space and enhanced diuresis with intravascular fluids; and (d) utilization of pharmacologic agents to counteract the nephrotoxic effects of contrast media [45].

For a long time, MM was considered a significant risk factor for developing contrast nephropathy [46]. This assumption was not lying on robust evidence-based data but only on early published studies that associated intravascular administration of contrast agent and kidney failure in patients with MM when using ionic agents [47, 48]. Those studies supported the idea that in patients with MM the intravascular administration of iodinated contrast media leads to the sum of two risk factors: the potential nephrotoxic action of contrast media added to the developing/developed kidney disease in malignant plasma cell dyscrasias [49]. As a consequence, radiologists appear to be very conservative in using iodinated intravascular contrast in patients with MM, even though the relative low incidence of CI-AKI in multiple myeloma patients was early reported to be approximately 0.6–1.25% compared to a risk of 0.15% in the general public [50] and more recently 5% within 48 hours and 15% within 7 days [51]. Several institutional practice guidelines and epidemiological studies [52] suggest that the presumed risk of CI-AKI can be stratified by using three serum creatinine levels: low risk, serum creatinine less than 1.5 mg/dL (<132.6 *μ*mol/L), medium risk 1.5–2.0 mg/dL (132.6–176.8 *μ*mol/L), and high risk more than 2.0 mg/dL (>176.8 *μ*mol/L). Data from a survey performed in 2006 showed that 36% of USA radiologists never administer intravascular contrast agent to patients with MM and 47% do it sometimes [53]; in addition, 56% of nonradiology physicians consider MM with normal renal function to place the patient at increased risk of adverse events associated with iodinated contrast agent administration [54]. Dehydration, infection, hypercalcemia, and Bence Jones proteinuria, which are conditions very often associated with MM, should be considered the primary risk factors for the development of CI-AKI in myeloma [55]. Therefore, there is a need to reconsider the role of intravascular contrast media as risk factor in patients with malignant plasma cell dyscrasia. Several guidelines, published by groups of expert radiologists, nephrologists, and cardiologists, recommend recognizing patients at risk of developing CI-AKI before injection of intravascular contrast media [56–58]. Main risk factors have been reported to be CKD, diabetes, heart failure, low body mass index, hypotension, sepsis, and aging. However, results obtained in a recent meta-analysis lead to the conclusion that out of all risk factors mentioned in published guidelines only a few are significantly associated with contrast-induced nephropathy after intravenous iodinated contrast medium administration [59], namely, CKD, diabetes, aging above 65 years, and use of NSAIDs. Other risk factors such as hypertension, congestive heart failure, contrast volume, hydration status, and anemia do not appear to have a significant association with the incidence of contrast-induced nephropathy [59]. However, the role of anemia as a risk factor for developing CI-AKI should be reevaluated, especially in patients with multiple myeloma. A couple of very recent studies investigated the incidence of CI-AKI in anemic patients, suggesting that low hemoglobin and low hematocrit values may be considered independent predictors of CI-AKI [60, 61]. Since anemia is highly prevalent in multiple myeloma, being reported in about 70% of patients with newly diagnosed multiple myeloma [62, 63], it may represent an important risk factor. It is strongly recommended that risk assessment and prophylactic strategies should be based on eGFR rather than the absolute level of serum creatinine [64].

## 7. Diagnosis of Kidney Failure and Injury in Multiple Myeloma

The standard assessment of renal function in patients with MM includes serum creatinine and creatinine clearance, although both measurements probably underestimate the prevalence of renal dysfunction, because of the additional tubular secretion of creatinine and its dependence on extrarenal factors. In particular, serum creatinine is a retrospective, insensitive, and even deceptive measure of kidney injury. Retrospective because its concentration may result in a very delayed signal even after considerable kidney injury [65]. Insensitive because as much as a 50% loss of renal function may be required to elevate serum creatinine enough that it comes to medical attention, whereas levels that fall short of this threshold are usually dismissed, despite their known association with excess mortality and prolonged hospitalization. As serum creatinine is affected by tubular secretion and systemic production, changes in its concentration are not specific to tubular injury. Deceptive because serum creatinine level often reflects transient physiologic adaptations to volume changes or the presence of CKD rather than the development of AKI. The degree of kidney impairment in MM should be assessed following the Kidney Disease Improving Global Outcomes (KDIGO) classification [66]. In MM patients with stabilized serum creatinine, the International Myeloma Working Group (IMWG) recommended the modification of diet in renal disease (MDRD) equation for estimating GFR (eGFR) [4]. However, more recent studies are supporting the use of the chronic kidney disease epidemiology collaboration cystatin C-based equations (both CKD-EPI-sCR-CysC and CKD-EPI-CysC) [67]. CKD-EPI equations based on cystatin C detect more MM patients with stages 3–5 kidney failure than equations based only on serum creatinine, namely, the MDRD and the original CKD-EPI equations. Interestingly, CKD-EPI-CysC equation predicts overall survival. It is not surprising that the diagnosis of a paraproteinemic renal lesion is hampered by the general lack of sensitivity and specificity of currently available noninvasive tests. The gold standard for diagnosis remains renal biopsy with demonstrated evidence of deposition of monoclonal proteins in the area of injury [68]. The measurement of serum concentration of the clonal FLCs and that of urine level of albumin may be considered the landmarks for screening algorithms in patients with cast nephropathy [69]. It has been proposed that when the concentration of serum FLCs exceeds 500 mg/L and proteinuria is mainly composed of immunoglobulin light chains, it is extremely likely that the onset of AKI is due to myeloma kidney; in those cases, renal biopsy is usually unnecessary. On the other hand, significant amount of albumin in urine is frequently associated with amyloidosis or MIDD. Amyloid deposits may be confirmed on biopsy of subcutaneous fat or of one of the minor salivary glands. If both are negative, then a kidney biopsy is required to search for amyloid, MIDD, or an unrelated glomerulopathy such as diabetic nephropathy. Patients with CKD and a monoclonal protein need special care, being at increased risk of AKI; renal biopsy is necessary to determine the cause of kidney damage.

## 8. Novel Biomarkers of Toxic Nephropathy and Contrast-Induced Nephrotoxicity

Identification of novel toxic nephropathy and AKI biomarkers has been designated as a top priority by the American Society of Nephrology. The concept of developing a new toolbox for earlier diagnosis of disease states is also prominently featured in the National Institute of Health (NIH) road map for biomedical research. In 2007, the Acute Kidney Injury Network (AKIN), a collaborative group of investigators from all major critical care and nephrology societies, proposed a staging system based on serum creatinine and urine output and consisting of 3 categories (mild, moderate, and severe) in a way similar to those (risk, injury, and failure) used by the RIFLE staging system. AKIN criteria have been included and recommended in the recent AKI guideline published by the Kidney Disease: Improving Global Outcomes (KDIGO) [70]. Despite these working classification systems, the diagnosis of AKI is problematic, as current diagnoses rely on two functional abnormalities: functional changes in serum creatinine and oliguria. Both of these are late consequences of injury and not markers of the injury itself. Most importantly, the measurement of serum creatinine does not identify the cell type that is acutely injured, even though this localization determines the natural history of the disease and its response to therapy. Neutrophil gelatinase-associated lipocalin (NGAL) has emerged as the most promising marker of AKI in a number of clearly defined clinical contexts [71]. NGAL, also known as* lcn2,* is one of the most upregulated transcripts in the early postischemic mouse kidney and this finding has been confirmed in several other transcriptome profiling studies. Downstream proteomic studies have also revealed NGAL to be one of the earliest and most robustly induced proteins in the kidney after ischemic or nephrotoxic AKI in animal models, and NGAL protein is easily detected in the blood and urine soon after AKI [72]. These findings have spawned a number of translational proteomic studies to evaluate NGAL as a novel biomarker in human AKI. A second very promising biomarker of kidney damage is kidney injury molecule 1 (KIM-1), a type-1 transmembrane glycosylated protein with IgG-like domains in the ectodomain of the protein. Downstream proteomic studies have also shown KIM-1 to be one of the most highly induced proteins in the kidney after AKI in animal models, and a proteolytically processed domain of KIM-1 is easily detected in the urine soon after AKI [73]. KIM-1 represents a promising candidate for inclusion in the urinary “AKI panel” [74, 75]. An advantage of KIM-1 over NGAL is that it appears to be more specific to ischemic or nephrotoxic AKI and not significantly affected by prerenal azotemia, urinary tract infections, or chronic kidney disease. It is likely that NGAL and KIM-1 will emerge as tandem biomarkers of AKI, with NGAL being most sensitive at the earliest time points and KIM-1 adding significant specificity at slightly later time points. In a small human cross-sectional study, KIM-1 was found to be markedly induced in proximal tubules in kidney biopsies from patients with established AKI (primarily ischemic). However, patients with AKI induced by contrast media did not have significant increase in urinary KIM-1 concentration [76]. Additional promising biomarkers for the early diagnosis and management of CI-AKI may be the cysteine rich protein 61 (CYR61), induced in the kidney within one hour after ischemic/toxic injury and detectable in the urine 3–6 hours later [77], Krüppel-like factor 6 (Zf9), a Krüppel-like transcription factor involved in the regulation of a number of downstream targets [78], thrombospondin 1 (TSP-1), a p53-dependent proapoptotic and antiangiogenic molecule [79], and IL-18, a proinflammatory cytokine that is known to be induced and cleaved in the proximal tubule and subsequently easily detected in the urine following ischemic AKI in animal models [80]. New encouraging perspectives for improving the current state in clinical diagnosis and in the identification of nephrotoxic injuries are emerging from metabolomics studies [81]. Metabolomics aims to identify unique fingerprints of specific injuries and functions, for example, diseases or effects of exposure to toxic compounds [82]. Predictive models based upon proton nuclear magnetic resonance spectroscopy (^1^H NMR) of rodent urine and serum demonstrated that liver and kidney toxicity can be predicted with sensitivities of 41 and 67% and specificities of 100% and 77%, respectively [83].

## 9. Clinical Approach to a Patient with Plasma Cell Dyscrasia about to Undergo Radiocontrast Studies

Although plasma cell dyscrasia would not be “per se” risk factors for the occurrence of CI-AKI, when comorbidities are present, patients require to be carefully monitored after contrast media injection. Kidney impairment, a major risk factor for developing CI-AKI, is one of the more frequent clinical features of symptomatic multiple myeloma. Other coexisting morbidities include anemia, hypercalcemia, and potentially nephrotoxic drugs like NSAIDs, chemotherapeutic agents, and antibiotics. Often, multiple factors may coexist, like kidney impairment with anemia, infections with nephrotoxic drugs, diabetes with aging, and increasing the risk of CI-AKI [84]. The management of patients with plasma cell dyscrasia requiring the intravascular administration of iodinate contrast media should include (a) assessment of the risk before administration; (b) pharmacological and nonpharmacological preventive measures for reducing such risk factors; (c) monitoring after administration; (d) early accurate diagnosis of CI-AKI.

According to the 2012 KDIGO AKI guideline [70], before the intravascular administration of iodinate contrast media, it is mandatory to assess the population at risk by measuring serum creatinine together with the estimation of GFR by the CKD-EPI equation. It has been reported that the incidence of CI-AKI became significant from a baseline serum creatinine concentration of >1.8 mg/dL (>159 *μ*mol/L) [85]. However, the CI-AKI Consensus Working Panel agreed that the risk of CI-AKI becomes clinically important when the baseline serum creatinine concentration is ≥1.3 mg/dL (≥115 *μ*mol/L) in men and ≥1.0 mg/dL (≥88.4 *μ*mol/L) in women [86]. The guideline also recommends that precautions to reduce the risk should be implemented in patients with a baseline eGFR <45 mL/min per 1.73 m^2^ [70]. When a recent serum creatinine value is not available, a simple questionnaire (e.g., the Choyke questionnaire) [87] or a dipstick testing for urine protein may be useful for identifying preexisting kidney disease. However, dipstick testing is reliable for the identification of albuminuria but it is not able to identify Bence Jones proteinuria. Since serum *β*_2_-microglobulin increases with both higher tumor burden and diminished renal function, it was suggested that evaluation of *β*_2_-microglobulin levels may be useful before intravenous injection of iodinate contrast agents to patients with multiple myeloma [51]. Serum *β*_2_-microglobulin likely serves as a marker of patients who are at a higher risk of developing CI-AKI. According to previous results published elsewhere, a value of *β*_2_-microglobulin below 2.8 mg/L may be the most clinically useful marker for patients at low risk of developing CI-AKI, showing a 100% negative predictive value [51].

According to the 2012 KDIGO AKI guideline, pharmacological and nonpharmacological preventive measures for reducing risk factors for CI-AKI in patients with multiple myeloma include (a) the use of either iso-osmolar or low-osmolar rather than high-osmolar iodinated contrast media (strength of recommendation 1B); (b) extracellular volume expansion at the time of radiocontrast media administration with either isotonic sodium chloride or sodium bicarbonate solutions (strength of recommendation 1A). It is desirable not to use oral fluids alone (strength of recommendation 1C), while it is recommended to use oral* N*-acetylcysteine (NAC) together with intravenous isotonic crystalloids (strength of recommendation 2D). A meta-analysis on 41 studies showed that NAC is the most effective agent for preventing CI-AKI in patients with CKD [88]. Following evidence in animal model that aciduria increases the nephrotoxicity of Bence Jones protein independently of urinary flow rate [88, 89], alkalinization of the urine through oral bicarbonate has been recommended in patients with multiple myeloma and Bence Jones proteinuria to prevent or treat myeloma cast nephropathy. In a trial performed in myeloma patients presenting with renal failure, patients were randomized to receive either sodium bicarbonate to render their urine neutral or no supplement. Patients randomized to receive alkali fared marginally better than the others, but the difference was not significant [90]. Urine alkalinization is now recommended by consensus documents on the management of renal failure in multiple myeloma patients [4]. On the basis of recent evidence, it is reasonable to correct marked anemia in patients with mild-to-moderate kidney impairment before contrast studies. The combination of kidney disease with anemia significantly increases the risk of CI-AKI with reported incidences of 6.3–7.8% in anemic patients compared with 2.2–2.8% in nonanemic patients [60, 61]. When serum FLCs level exceeds 500 mg/L in patients with multiple myeloma and other lymphoproliferative disorders, it is reasonable to achieve a rapid reduction in serum FLCs levels before administrating the contrast medium [25]. This emergency treatment is based on high-dose dexamethasone. Bisphosphonates are effective for controlling malignancy-related hypercalcemia; nevertheless, they can further impair renal function and cause symptomatic hypocalcemia in patients with acute renal failure and their use is discouraged in such patients [91]. Although the reduction of hypercalcemia has been considered optional in the 2012 KDIGO AKI guideline, we suggest correcting high concentrations of serum calcium before contrast media studies, being a risk factor for acute kidney impairment. Finally, as a preventive measurement in multiple myeloma patients at high risk for CI-AKI, it is desirable to discontinue the therapeutic administration of NSAIDs, aminoglycosides, high dose diuretics, antineoplastic drugs, and metformin 48 hours before contrast media administration.

Multiple myeloma inpatients monitoring consists of measurement of serum creatinine together with the calculation of eGFR at 12 and 48 hours after contrast media injection. If there is evidence of CI-AKI, it is recommended to repeat these tests on days 3–5 after contrast media administration, monitoring also urine output, and it is reasonable to prolong the discontinuation of the therapeutic treatment with NSAIDs, aminoglycosides, high dose diuretics, antineoplastic drugs, and metformin [70].

Finally, diagnosis of CI-AKI can be improved by adding the measurement of serum cystatin C, which allows for an earlier assessment of changes in renal filtration [92]. Urine NGAL may be also useful for assessing the presence of kidney injury and damage, often preexistent in patients treated with nephrotoxic drugs.

## 10. Conclusions

Data from recent meta-analyses have led to the conclusion that the overall incidence of CI-AKI is low, not exceeding 6.4%; moreover, an association between the presence of malignancy with contrast-induced nephropathy was found [59, 93]. Nevertheless, this could result from comorbidities and the nephrotoxic chemotherapy administered to these patients in combination with intravenous iodinated contrast medium. Further evidence suggests that the incidence of CI-AKI might be overestimated. In a retrospective study on more than 50,000 patients divided in two subgroups (intravenous contrast-enhanced and contrast-unenhanced CT scan) it was found that the incidence of CI-AKI was not significantly different from AKI caused by other factors [94], confirming similar results published previously [95]. The hypothesis that CI-AKI might be overestimated was further supported by a meta-analysis including 13 studies and representing 25,950 patients; the incidence of CI-AKI was 6.4% (ranging from 2.1% to 19%), while the incidence of noncontrast medium AKI was 6.5% (ranging from 1.3% to 19.8%) [96].

So far, there is limited evidence that myeloma per se may be a risk factor for developing CI-AKI. A careful medical history examination together with serum creatinine and the estimation of GFR by using the equation CKD-EPI-sCr-Cys may be considered the best cost/effective strategy to prevent CI-AKI in patients with multiple myeloma. The use of NGAL and KIM-1 as tandem markers for early detection and later confirmation, respectively, of kidney injury in patients at high risk should be considered. When serum FLCs level exceeds 500 mg/L in patients with multiple myeloma and other lymphoproliferative disorders, it is reasonable to achieve a rapid reduction in serum FLCs levels before administrating the contrast medium. Additional preventive measures include the use of the lowest volumes required and the removal of concomitantly administered nephrotoxic substances/drugs, especially NSAIDs. We should avoid redundant laboratory tests, like Bence Jones proteinuria, since this test cannot be considered as a surrogate biomarker of kidney function.

## Figures and Tables

**Table 1:** Kidney disease in plasma cell dyscrasias ([14, 18]).

(I) Common	
(1) Light-chain cast nephropathy (myeloma kidney)	
(2) Immunoglobulin-related amyloidosis (AL, AHL, and AH)	
(3) Monoclonal immunoglobulin deposition disease (LCDD, LHCDD, and HCDD)	
(4) Acute tubular necrosis	
(A) Drugs (nonsteroidal anti-inflammatory drugs and bisphosphonates)	
(B) Intravascular iodinated contrast	
(5) Type I and type II cryoglobulinemic glomerulonephritis	
(II) Uncommon	
(1) Light chain proximal tubulopathy (with or without Fanconi syndrome)	
(2) Crystal-storing histiocytosis	
(3) Nonamyloid monoclonal fibrillary glomerulonephritis	
(4) Immunotactoid glomerulonephritis/glomerulonephritis with organized microtubular monoclonal immunoglobulin deposits (GOMMID)	
(5) C3 glomerulonephritis associated with monoclonal gammopathy	
(6) Proliferative glomerulonephritis with monoclonal Ig deposits	
(7) Hyperviscosity syndrome	
(A) Waldenström's macroglobulinemia	
(B) IgM, IgA, and rarely IgG myeloma	
(8) Plasma cell infiltration	
(9) Pyelonephritis	
(10) Uric acid nephropathy	

**Table 2:** Main risk factors for acute kidney injury (AKI) or acute tubular necrosis (ATN) in patients with malignant plasma cell dyscrasias.

(1) Comorbidities: chronic kidney disease, diabetes, aging, hypertension, and cardiovascular disease	
(2) Volume depletion (e.g., dehydration, etc.)	
(3) Hypercalcemia	
(4) Hyperuricemia	
(5) Repeated iodinated contrast media administration	
(6) Nonsteroid anti-inflammatory drugs	
(7) Diuretics	
(8) Aminoglycosides	
(9) Hyperviscosity syndrome	
